# A meta-analysis revealed insights into the sources, conservation and impact of microRNA 5′-isoforms in four model species

**DOI:** 10.1093/nar/gkt967

**Published:** 2013-10-30

**Authors:** Jing Xia, Weixiong Zhang

**Affiliations:** ^1^Institute for Systems Biology, Jianghan University, Wuhan, Hubei 430056, China, ^2^Department of Computer Science and Engineering, Washington University in Saint Louis, One Brookings Drive, St. Louis, MO 63130, USA and ^3^Department of Genetics, Washington University School of Medicine, St. Louis, MO 63110, USA

## Abstract

MicroRNA (miRNA) 5′-isoforms, or 5′-isomiRs, are small-RNA species that originate from the same genomic loci as the major miRNAs with their 5′ ends shifted from the 5′ ends of the miRNAs by a few nucleotides. Although 5′-isomiRs have been reported, their origins, properties and potential functions remain to be examined. We systematically studied 5′-isomiRs in human, mouse, fruitfly and worm by analysing a large collection of small non-coding RNA and mRNA profiling data. The results revealed a broad existence of 5′-isomiRs in the four species, many of which were conserved and could arise from genomic loci of canonical and non-canonical miRNAs. The well-conserved 5′-isomiRs have several features, including a preference of the 3p over the 5p arms of hairpins of conserved mammalian miRNAs, altered 5′-isomiRs across species and across tissues, and association with structural variations of miRNA hairpins. Importantly, 5′-isomiRs and their major miRNAs may have different mRNA targets and thus potentially play distinct roles of gene regulation, as shown by an integrative analysis combining miRNA and mRNA profiling data from psoriatic and normal human skin and from murine miRNA knockout assays. Indeed, 18 5′-isomiRs had aberrant expression in psoriatic human skin, suggesting their potential function in psoriasis pathogenesis. The results of the current study deepened our understanding of the diversity and conservation of miRNAs, their plasticity in gene regulation and potential broad function in complex diseases.

## INTRODUCTION

MicroRNAs (miRNAs) are ∼22-nt long small non-coding RNAs (sncRNAs) that play an important role of post-transcriptional gene regulation via transcript degradation and translation repression (1). The maturation of miRNAs involves multiple steps, during which two intermediate forms of miRNAs, primary (pri-) and precursor (pre-)miRNAs, are produced sequentially. In this process, two RNase III enzymes, Drosha and Dicer, cleave pri- and pre-miRNAs consecutively to release ∼22-nt double-stranded RNAs with ∼2-nt 3′ overhangs, namely miRNA/miRNA* duplexes. The mature miRNAs are then loaded into Argonaute proteins to exert their regulatory effect by binding to target transcripts (2).

The most abundant miRNA sequences from pre-miRNA hairpins are normally used to define the corresponding miRNAs. Nevertheless, less abundant, cognate variant sequences from the same pre-miRNAs have been identified in early cloning studies, which are often discarded (3,4). The variation in the positions of miRNAs’ termini was regarded as heterogeneity of miRNAs. With more miRNA heterogeneities being detected by deep sequencing, the term *isomiR* has been introduced to recognize cognate miRNA variants (5). isomiRs have been observed in various organisms, such as human (*Homo sapiens*), mouse (*Mus musculus*), fruitfly (*Drosophila melanogaster*), worm (*Caenorhabditis elegans*), plants and viruses (6–17). The authenticity of isomiRs as genuine miRNAs have been supported by several lines of evidence, including detection using both linker-based miRNA cloning and massively parallel sequencing as well as the fact that they can be loaded into Argonautes (3,5,6).

Two types of isomiRs—5′-isomiRs and 3′-isomiRs—exist, where the 5′ and 3′ termini of miRNA isoforms, respectively, are shifted from that of their major miRNAs by a few nucleotides. 3′-isomiRs are more prevalent than 5′-isomiRs and have been reported to influence miRNA stability and efficiency of target repression (18–20). On the other hand, 5′-isomiRs have different seed regions from the major miRNAs; it is known that miRNA seed regions, which span across the second to the seventh nucleotides of the 5′ termini of miRNAs, are key determinants of the specificity of miRNA and target binding (2,21,22). Therefore, 5′-isomiRs are more likely to have a direct effect on gene regulation.

Diverse mechanisms may underlie the generation of 5′-isomiRs. A recent study has shown that *Drosophila* Dicer-1, in association with various alternatively spliced isoforms of Dicer partner protein, *loquacious* (*loqs*), is capable of adjusting the length of mature miRNAs on pre-miR-307a, leading to the generation of 5′-isomiRs with distinct target specificity (23). In human, Dicer also has variable cleavages based on some previously described counting rules of 5′/3′ precursor ends (24). A more recent study has discovered additional loop-counting rules based on pre-miRNA structures. It shows that the inaccurate Dicer cleavages are abrogated when 5′ ends on 3p arms of pre-miRNAs are positioned 2-nt away from the nearest upstream bulges or loops (25). Independent of Dicer, some isomiRs potentially derive from AGO2 cleavage and associate with translational machinery (26).

Non-canonical miRNAs are generated from alternative biogenesis pathways where Drosha, a key player in the canonical miRNA biogenesis pathway, is typically not involved. For example, miRtrons are generated from debranched introns of protein-coding genes; therefore no Drosha cleavage activities are needed (27). Recent studies have also reported that specific human snoRNAs (e.g. human ACA45) and a murine tRNA (i.e. murine tRNA-Ile) can produce miRNA-like RNAs (28,29). These miRNA-like RNAs are found to be independent of Drosha and arise from local hairpin formation within larger non-coding RNA (ncRNA) species, such as snoRNAs (16,28,30). In the case of the murine tRNA-Ile, the transcript can fold into alternative secondary structures in form of miRNA precursors which bypass Drosha processing (29). Of particular interest are the non-canonical miRNAs and miRNA-like RNAs expressed in human skin (31), which will be further analysed for isomiR production in the current study.

Several lines of evidence indicate that similar to major miRNAs, 5′-isomiRs are capable of targeting mRNAs and play functional roles different from the major miRNAs (8,26,32–34). Despite prevalence of 5′-isomiRs, their function remains to be systematically investigated. Here, we collected a large set of profiling data of sncRNAs and protein-coding genes from multiple sources for a meta-analysis of 5′-isomiRs, i.e. we performed a comprehensive study of 5′-isomiRs in four model species, human (*H. sapiens*), mouse (*M. musculus*), fruitfly (*D. melanogaster*) and worm (*C. elegans*). Our emphasis was on human 5′-isomiRs using our recently collected small-RNA deep-sequencing data from 67 psoriatic and normal human skin biopsy samples (31,35). To further investigate the potential functions of 5′-isomiRs, we also analysed gene-profiling data from murine miRNA knockouts carrying 5′-isomiRs. Our results provided insights into the sources, conservation and potential functions of 5′-isomiRs.

## MATERIALS AND METHODS

### Small-RNA datasets and preprocessing

A total of 132 small-RNA sequence libraries with more than 816 million sequencing reads were analysed. These small-RNA datasets were collected from four species including human (*H. sapiens*), mouse (*M. musculus*), fruitfly (*D. melanogaster*) and worm (*C. elegans*). The datasets on human include 67 libraries from psoriatic and normal human skin, 4 libraries from human stem cells and 2 libraries from human brain; the mouse datasets consist of 42 libraries from skin, neutrophil, dendritic, brain, ovary, testes and embryonic stem cells, three embryonic stages, and whole newborn mice; the fruitfly datasets comprise 7 libraries from ovary, head and S2 cells; the worm datasets contain 10 libraries from embryo and whole body (Supplementary Table S1A). When available, 3′ adaptor-trimmed qualified reads were obtained from GEO databases; otherwise, raw sequencing reads were processed for 3′ adapter stripping using an in-house program (35). Bowtie (36) was used to map the processed reads to miRNA hairpins with zero mismatches. Information of mature miRNAs in the four species and the sequence of their precursors and hairpins were obtained from miRBase version 18 (37).

### Analysis of miRNA 5′ variations

Qualified reads of different datasets mapped to miRNA hairpins were separated into the 5p and 3p arms of each individual miRNA hairpin. The mature miRNA on one arm of a hairpin annotated in miRBase was considered as the major miRNA for the arm. The sequences on one arm whose 5′ ends shifted from the 5′ end of the major miRNA by no more than 5 nt were taken as 5′-isomiRs. The number of reads for a 5′-isomiR derived from a set of libraries (e.g. from human skin or mouse brain) was considered as the abundance of the 5′-isomiR. To measure the degree of heterogeneity of a miRNA on an arm of a miRNA hairpin, the *arm abundance* of the 5′-isomiR was introduced and defined as the ratio of the number of reads for the 5′-isomiR to the total number of reads mapped to the arm.

### Analysis of 5′-isomiR conservation

We followed the miRNA annotation in miRBase (version 18) (37) where miRNA orthologs were given the same family number across species. Two miRNAs were considered as orthologs if they had nearly identical mature miRNAs and similar hairpin sequences (38). This annotation allowed us to identify orthologous miRNAs well-conserved in mammal, hexapoda and nematoda.

To find miRNAs conserved in mammal, miRNA genes in human and mouse were obtained from miRBase. miRNAs having the common family names in the two species were considered to be conserved in mammal. Similar analyses were carried out to identify miRNAs conserved in hexapoda and nematode. Common miRNA family names between *Apis mellifera* and *D. melanogaster* were considered as conserved miRNAs in hexapoda, and common names in *Haemonchus contortus* and *C. elegans* were treated as conserved miRNAs in nematoda. Finally, expressed 5′-isomiRs from orthologous miRNAs were compared. Sequences with the same or nearly identical seed regions, with no more than 1 nt mismatch, were considered as conserved 5′-isomiRs.

### Comparisons of arm abundances across species and across tissues

Arm abundances of 5′-isomiRs across species were compared to analyse 5′-isomiR conservation. This was done by comparing arm abundances of 5′-isomiRs in the same (or similar) tissues or organs across species (black arrows in Supplementary Figure S1). The analysis of tissue specificity of 5′-isomiRs was performed across tissues in one species (red arrows in Supplementary Figure S1). Moreover, tissue specificities in human and mouse (blue arrows in Supplementary Figure S1) were also analysed in order to discover the conservation of tissue specificities.

### Analysis of structural conservation

The structure information of pre-miRNA sequences in the four species was obtained from miRBase (version 18). Consensus secondary structures of conserved pre-miRNAs were predicted by ClustalW (39) and RNAalifold in the Vienna package (40). RNAalifold searches for conserved secondary structures rather than conserved sequences. For example, two RNA sequences with little identical sequences can have a low consensus value (i.e. a high conservation) as long as they contain a similar type of secondary structure such as a hairpin.

### Differential expression of 5′-isomiRs

The abundance of a 5′-isomiR was first determined by the number of reads that aligned perfectly to the 5′-isomiR sequence. Reads that mapped to multiple genomic loci were attributed to all potential derivative small RNAs. The abundance of 5′-isomiRs in number of reads are then normalized to adjust for variation across different libraries or conditions. As no consensus method is currently available for normalizing miRNA profiling data (41), following our previous studies (31,35,42,43) we assumed the overall abundance of all sncRNAs, including miRNAs and various siRNAs, remained a constant in the cell in our normalization method. Thus the abundance of a 5′-isomiR in a library (condition) was normalized by multiplying a constant factor of *C*/*T*, where *T* is the total number of reads that were mapped to the reference genome (e.g. human genome hg19 build) in the library being analysis and *C* is a large constant, say 1 million, or the average of total reads of every library considered that were mapped to the reference genome. Fold changes of 5′-isomiRs across conditions were calculated from normalized read counts, and chi-squared test was applied to determine their statistical significance.

### miRNA target prediction

Two widely adopted miRNA target prediction methods, TargetScan (version 5.2) (44) and miRanda (45), were used to predict the targets of miRNAs and isomiRs. miRanda is less sensitive to seed regions since it uses sequence information of seed region and mature sequence of a miRNA. Putative targets of miRNAs and companion 5′-isomiRs were first predicted using TargetScan and those targets that were also predicted by miRanda were then removed from the target set of 5′-isomiRs to form the set of genes exclusively targeted by 5′-isomiRs. The common targets between miRNAs and 5′-isomiRs were predicted by both miRanda and TargetScan. This stringent miRNA target prediction criterion eliminated or reduced the false-positives in the set of putative target genes exclusively targeted by 5′-isomiRs. The common and 5′-isomiR’s exclusive targets were grouped into the 7mer-A1, 7mer-m8 and 8mer categories of miRNA-binding sites (2) according to the appearance of these motifs within the target sites for further analysis. Briefly, a 7mer-A1 site on a mRNA transcript has an exact match to the seed region, i.e. positions 2–7 of a miRNA, followed by an ‘A’; a 7mer-m8 site has an exact match from position 2 to position 8 of a miRNA; an 8mer site is a 7mer-m8 followed by an ‘A’ (2). Some mRNAs not targeted by a miRNA or the associated 5′-isomiR were randomly chosen to form the set of *no-site* mRNAs as a control for the analysis of the exclusive targets of the 5′-isomiR (see the detail and a statistical analysis in the next section).

### Analysis of microarray mRNA gene expression from miRNA knockout assays

Microarray datasets of mmu-miR-223 and mmu-miR-142 knockout experiments (GSE22004, GSE42325, Supplementary Table S1B) were collected in recent studies (21,46). The fold change of a gene was calculated using the expression levels before and after deleting a miRNA. Genes were categorized into targets for 8mer, 7mer-m8 and 7mer-A1 miRNAs or non-targets (no site) according to target prediction. A random sampling on the non-target genes was applied to generate a control set with the same size as the target set. In addition, features of similar expression level and 3′UTR length were taken into consideration in generating control sets. As a result, we constructed a control set from the non-target genes that had expression levels and 3′UTR lengths similar to that of the target genes. Specifically, the expression levels of a sampled gene and a target gene differ no more than 20 raw signal value from array datasets, and their 3′ UTR lengths differ no more than 50 nt.

One-sided Kolmogorov–Smirnov (K-S) test was adopted to compare the target sets of 8mer, 7mer-m8 and 7mer-A1 separately against the control set. The *P*-value from the K-S test was used to measure the significance of a comparison. A smaller *P*-value from K-S test indicates a more significant difference between the two tested distributions. The K-S statistic has the advantages that it is non-parametric in that it makes no assumption on the underlying distribution of expression changes and it can deal with continuous distributions. The K-S statistic computes the maximum difference between two empirical cumulative distribution functions, which constitute fold-change values of mRNA expressions.

### Analysis of microarray mRNA gene expression from human psoriatic and normal skin

Microarray data from 58 psoriatic patients and 64 normal healthy controls were retrieved from NCBI/GEO databases (accession number GSE13355, Supplementary Table S1B). The expression levels were normalized using the robust multichip average method (47), and normalized expression levels were used for identifying differentially expressed (DE) genes using Rank Product (48). It has been shown that Rank Product is less sensitive to noise and has a better performance than other methods when sample size is small (48). Probes with False Positive rate, equivalent to False Discovery Rate, no >0.05 for 1000 permutations were taken as DE. DE probes were then mapped to corresponding genes according to the annotation of Affymetrix Human Genome U133 Plus 2.0 Array.

Pairs of anti-correlated 5′-isomiRs and targeted mRNAs were identified if DE 5′-isomiRs and their DE target genes exhibited anti-correlated relationship. We constructed control sets of the same size as the DE 5′-isomiR set by randomly sampling from non-DE miRNAs and 5′-isomiRs. We predicted their target genes by TargetScan and counted the number of DE mRNAs among these predicted targets. Statistical *P*-values were calculated based on a T-test.

### GO analysis of targets of miRNAs and 5′-isomiRs

Gene Ontology (GO) term analysis was performed using the online tool DAVID (49). DAVID finds enriched GO terms for a given set of genes and performs a modified Fisher’s exact test on the GO terms to assess their biological significance.

## RESULTS

As detected by the large collection of sequencing data (see Materials section), more than 70% of the expressed miRNA hairpins in human, mouse, fruitfly and worm produced reads representing 5′-isomiRs (Table 1). Specifically, the hairpins of 1117 (82% of the total) miRNAs in human, 605 (89%) miRNAs in mouse, 162 (72%) miRNAs in fruitfly and 133 (71%) miRNAs in worm were recognized to produce 5′-isomiRs, showing that 5′-isomiRs widely exist in the animal kingdom (Table 1 and Supplementary Tables S2, S3, S4 and S5). Although such a large number of miRNAs yielded 5′-isomiRs, the amount of 5′-isomiR reads constituted a small portion of the total miRNA reads overall, i.e. they represented only 6.3, 8.3, 3.8 and 0.53% of the total sequencing reads in human, mouse, fruitfly and worm, respectively (Table 1).

**Table 1.** Summary of expressed miRNA hairpins that produce 5′-isomiRs in the four species analysed

Species	No. of expressed miRNAs	No. of miRNAs with 5′-isomiRs	Percentage of expressed miRNAs^a^	Percentage of overall miRNA reads^b^
Human	1353	1117	82.6	6.32
Mouse	673	605	89.9	8.36
Fruitfly	224	165	73.7	3.86
Worm	188	135	71.8	0.53

^a^Percentage of the total expressed miRNAs that produce 5′-isomiRs.

^b^Percentage of the total miRNA reads that represent 5′-isomiRs.

Importantly, miRNAs from the 3p arms of miRNA precursors in human and mouse had an overall higher degree of heterogeneity than from the 5p arms, consistent with the previous results (34). In human, 12.29% and 1.78% of the total reads mapped to the 3p and the 5p arms of hairpins, respectively, contributed to 5′-isomiRs in human skin (Supplementary Table S6), showing a ∼7-fold difference between the two. Beyond human, mouse had a similar overall 5′-isomiR preference as in human (11.79% versus 5.35% on the 3p and the 5p arms, respectively), whereas fruitfly and worm*,* however*,* had smaller differences between 5′-isomiRs on the 3p and the 5p arms of miRNA hairpins (4.2% versus 2.7% in fruitfly and 0.4% versus 0.5% in worm, Supplementary Table S6), consistent with the recent study of miRNA isoforms in fruitfly (7).

### Absolute and arm abundances of 5′-isomiRs

Individual miRNA hairpins in the four species carried variable amounts of 5′-isomiRs, ranging from millions to a few sequencing reads. Despite their relatively low levels of overall expression, there existed highly expressed individual 5′-isomiRs. Table 2 lists the top 10 most abundant 5′-isomiRs ranked by their read counts in human skin. Remarkably, the 3p arm of miR-203 hairpin carried the most abundant 5′-isomiRs, which ranked the 8th of all major miRNAs and miRNA isoforms expressed in human skin (Table 2). The let-7a family also produced 269,329 5′-isomiR reads from its 5p arm (Table 2), more abundant than ∼222K of the median number of reads of major miRNAs, suggesting that abundant 5′-isomiRs may function as well as those moderately expressed major miRNAs.

**Table 2.** The top 10 most abundant 5′-isomiRs in human skin

miRNA family	Seed	Arm	Rank^a^	No. of 5′-isomiR reads	Arm abundance
hsa-miR-203	GAAAUGU	3p	8	8,774,451	45.5
hsa-miR-140	CCACAGG	3p	37	1,072,944	39.5
hsa-miR-126	GUACCGU	3p	54	776,502	13.8
hsa-miR-199b	ACAGUAG	3p	55	724,009	27.0
hsa-miR-101	UACAGUA	3p	57	662,855	27.3
hsa-miR-10a	CCCUGUA	5p	59	661,921	22.7
hsa-miR-143	UGAGAUG	3p	69	619,911	1.8
hsa-miR-378a	UGGACUU	3p	71	402,197	6.0
hsa-miR-29a	UAGCACC	3p	77	334,605	18.1
hsa-let-7a	AGGUAGU	5p	89	269,329	0.6

The arm abundance of a 5′-isomiR is the percentage of all reads mapped to one arm that represents the 5′-isomiR. Seed region represents the range from 2 nt to 8 nt of a 5′-isomiR sequence.

^a^Rank of the 5′-isomiR among all miRNAs including major miRNAs, determined by the number of sequencing reads.

Besides variant absolute abundances of 5′-isomiRs, 5′-isomiRs’ abundance relative to that of their major miRNAs varied as well. For example, the abundance of the 5′-isomiRs from miR-203-3p accounted for 45.5% of the total miR-203-3p abundance whereas the abundance of the 5′-isomiRs from let-7a-5p was only 0.61% of the total let-7a-5p abundance. In order to properly measure the degree of 5′ end heterogeneity, we introduced *arm abundance* of a 5′-isomiR on one arm of a hairpin as the percentage of all reads of the arm that represent the 5′-isomiR.

**Figure 1.**
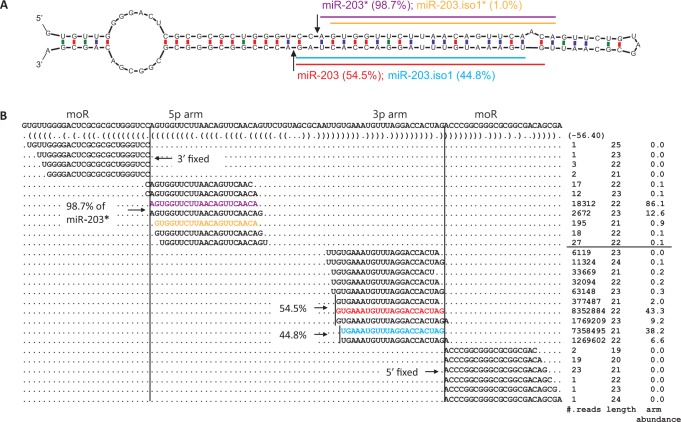
(**A**) miR-203 hairpin with 5′-isomiRs and the major miRNA expressed in human skin. The Drosha cleavage site is shown by two arrows. The horizontal color lines indicate the annotated major miR-203 (red), and its less abundant 5′-isomiR, miR-203.iso1 (blue), on the 3p arm. Ratios of reads for 5′-isomiRs over the total number of reads mapped to the arm (arm abundance) are shown in parentheses. (**B**) Read alignments represent 5′-isomiRs, as colored in (A). The vertical lines correspond to the Drosha cleavage site. When counting a 5′-isomiR, reads of the same 5′ terminal are grouped together. The flanking reads represent offset miRNAs (moR).

5′-isomiRs can have different arm abundances on the 3p and 5p arms of miRNA hairpins, reflecting different degrees of 5′ end heterogeneity of miRNAs between the two hairpin arms. One illustrating example is the hsa-miR-203 hairpin (Figure 1), where the arm abundance of 5′-isomiRs on the 3p arm was more than 40% versus 1.3% on the 5p arm. This is further illustrated by the two sharp boundaries separating major miR-203 and the flanking reads, indicated by the arrows in Figure 1A and the two vertical lines in Figure 1B. The 3p arm of murine mmu-miR-203 hairpin also had a higher 5′-isomiR arm abundance (34.9%) than the 5p arm (2%) (Supplementary Table S3), suggesting that the mechanism of yielding 5′-isomiRs of miR-203 is conserved in mammals. In addition, 5′-isomiRs on 39 out of 320 (12.2%) well-conserved miRNA hairpins had arm abundances at least 2-fold greater on the 3p arms than on the 5p arms (chi-square *P*-value < 0.01, Supplementary Table S7). In such cases, a low 5′-hetegenontiy on the 5p arms indicated a high Drosha fidelity, and a high 5′-hetegenontiy on the 3p arms implied an inaccurate Dicer processing.

### Broad existence and conservation of 5′-isomiRs

Well-conserved miRNAs produced 5′-isomiRs (Table 3), implying a ubiquitous presence and broad conservation of 5′-isomiRs in animals. Among the 221 miRNA families conserved in human and mouse, 185 (84%) produced 5′-isomiRs with the same seed regions (Supplementary Table S8). One such example is the miR-10 family where 5′-isomiRs of the same seed ‘CCCUGUA’ contributed to 21.8% and 28.6% of the miR-10a-5p abundances in human and mouse, respectively. Moreover, more than 50% of the conserved mammalian hairpins had high arm abundances of 5′-isomiRs (>10%) in multiple tissues or cell lines (Supplementary Tables S2 and S3). For example, miR-142 had a high 5′-heterogenity among all the mammalian small-RNA datasets that were examined, indicating a ubiquitous expression of 5′-isomiRs from miR-142 hairpin (Supplementary Tables S2 and S3).

**Table 3.** Well-conserved miRNA families that produce 5′-isomiRs and their seed regions

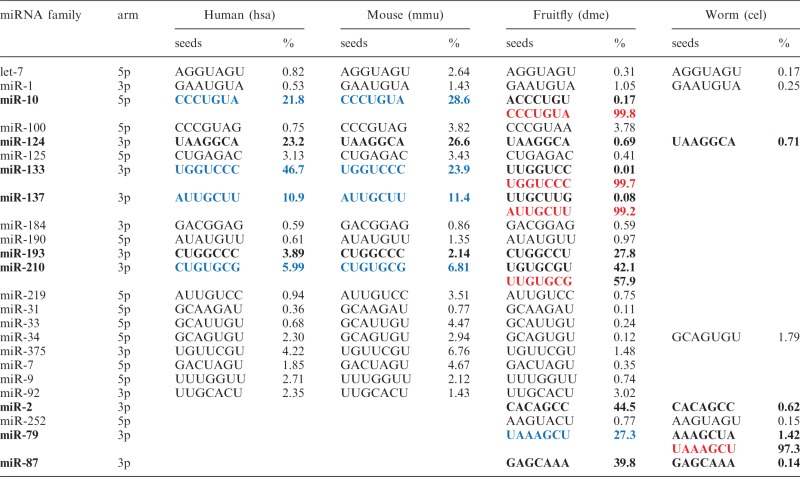

In the table, percentage (%) is the maximum of arm abundances among family members in an organism. miRNA families having significantly different arm abundances of 5′-isomiRs across species are shown in bold. 5′-isomiRs highlighted in blue switch to major miRNAs highlighted in red across species.

Beyond mammals, 20 miRNA families were found conserved across fruitfly, mouse and human, 8 were conserved between fruitfly and worm, and 4 families (let-7, miR-1, miR-34 and miR-124) were found conserved among all the 4 species. Besides well-conserved 5′-isomiRs, species-specific 5′-isomiRs exist in each of these species. The most prominent examples include hsa-miR-944 in human (14% of reads correspond to miR-944 5′-isomiRs, Supplementary Table S2), mmu-miR-295 in mouse (24%, Supplementary Table S3), dme-miR-994 in fruitfly (38%, Supplementary Table S4), which has been previously annotated as a prominent miRNA with highly imprecise 5′ ends (7).

### Characteristics of 5′-isomiRs

#### isomiR/isomiR* duplexes and arm preferences

Similar to miRNA/miRNA* duplexes, 5′-isomiRs may exist on both arms of a miRNA hairpin and form a paired duplex with a ∼2-nt 3′ overhang, which is reminiscent of RNase III enzyme processing (Figure 1A) and further authenticates isomiRs as genuine miRNAs. Following the nomenclature of miRNA and miRNA*, we named such a pair of small RNAs 5′-isomiR and 5′-isomiR* and added suffixes of iso1 and iso1* accordingly (Figure 1A). Dominance of a 5′-isomiR on one arm over the other of a miRNA hairpin was prominent, exemplified by the 5′-isomiR of hsa-miR-203 (Figure 1B), where more than 8 million reads were from the 3p arm (hsa-miR-203.iso1) versus ∼200 reads from the 5p arm (hsa-miR-203.iso1*).

**Figure 2.**
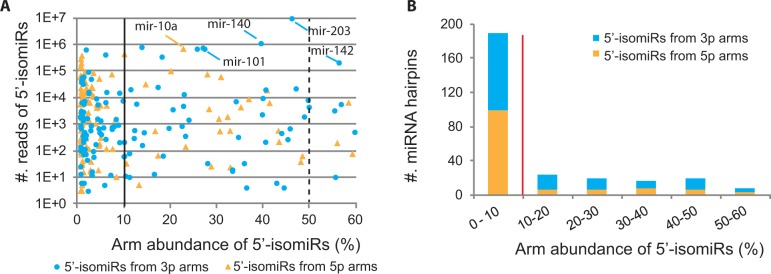
(**A**) Arm abundances of 5′-isomiRs from human miRNA hairpins conserved in mammal. The numbers of 5′-isomiR reads and arm abundances were derived from human psoriasis data. Dominant 5′-isomiRs derived from the 3p or 5p arms of miRNA hairpins are indicated in blue and orange, respectively. The solid line separates miRNA hairpins with 5′-isomiRs having arm abundances >10% from that <10%, and the dashed line indicates 5′-isomiRs with greater arm abundances than their major miRNAs. (**B**) The distribution of miRNAs with human 5′-isomiRs conserved in mammal over their arm abundances. The same color scheme as in (A) is used, showing that hairpins with dominant 5′-isomiRs having arm abundances >10% (the right side of the red line) appear more often on the 3p arms than on the 5p arms.

A substantial number of the well-conserved human miRNA hairpins carried 5′-isomiRs with <10% arm abundances in human skin (Figure 2). The rest human miRNA hairpins, which had 5′-isomiRs with more than 10% arm abundances, had nearly 2-folds more dominant 5′-isomiRs from the 3p arms than the 5p arms, i.e. 59 from the 3p arms versus 29 from the 5p arms (Table 4). Dominant 5′-isomiRs also appeared more frequently from the 3p arms than the 5p arms for well-conserved miRNAs in mouse, i.e. 59 from the 3p arms versus 30 from the 5p arms (Table 4), indicating that the preference for the 3p arms over the 5p arms is well preserved for mammalian miRNAs with high 5′-heterogeneities. 5′-isomiRs’ preference of favoring the 3p over 5p arms persisted in well-conserved miRNAs in fruitfly and worm (Table 4).

**Table 4.** Property of miRNA hairpins with more than 10% arm abundance of 5′-isomiRs

	Human			Mouse			Fruitfly			Worm		
	*C*^a^	*N*	%	*C*^a^	*N*	%	*C*^b^	*N*	%**^a^**	*C*^c^	*N*	%
*Hairpins with dominant 5′-isomiRs (Arm abundance >10%)*												
Arise from 5p	29	190	40	30	77	39	2	10	20	0	6	13
Arise from 3p	59	199	47	59	89	53	12	21	53	2	16	39
Arise from both arms	5	65	13	3	20	8	5	12	27	1	21	48

In the table, ‘*C*’ represents the miRNA hairpins conserved in a specified species class, and ‘*N*’ represents non-conserved miRNA hairpins. Percentage (%) is the proportion of the miRNA hairpins (sum of conserved and non-conserved).

^a^Conserved in mammal.

^b^Conserved in hexapoda.

^c^Conserved in nematoda.

#### 5′-isomiRs from orthologous miRNAs across species

Remarkably, major miRNAs and 5′-isomiRs may swap in different species. For example, miR-10-5p.iso1 in fruitfly is the major miR-10a-5p in human and mouse (miRBase, Supplementary Figure S2). Furthermore, miR-10a-5p.iso1 in human and mouse had a high arm abundance, whereas miR-10-5p.iso1 in fruitfly were slightly expressed (Table 3). Such seed shift, as previously reported (50), was also identified in miR-133-3p and miR-137-3p across fruitfly and human/mouse (Table 3), and found in miR-79-3p between fruitfly and worm.

Besides shifted seeds, many well-conserved 5′-isomiRs with the same or nearly identical seed regions had different arm abundances among the four species, exemplified by miR-124, miR-193, miR-210, miR-2 and miR-87 (Table 3). For instance, miR-124-3p family produced 22% and 27% of reads as 5′-isomiRs in human and mouse, respectively, but only the maximum of 0.69% of reads in fruitfly. In addition, dme-miR-210-3p had nearly 57.8% of reads as 5′-isomiRs in fruitfly while only 6.0% and 0.68% in human and mouse, respectively (Table 3). Such variations were also observed for miRNAs conserved only in fruitfly and worm, such as miR-2-3p and miR-87-3p (Table 3). Taken together, well-conserved miRNA hairpins can have dramatically different arm abundances of 5′-isomiRs across species.

#### 5′-isomiRs from paralogous miRNAs in human

Among the 221 mammalian conserved human miRNA families, 93 (42%) have multiple individual members, e.g. the let-7 family has 8 members. Two conserved miRNA families with multiple members, i.e. miR-133 and miR-10, had individual members with large differential 5′-isomiR arm abundances. For example, human miR-133a-1 and -2 together had an overall 47% of sequencing reads on the 3p arms as 5′-isomiRs whereas miR-133b-3p had only 10% of reads as 5′-isomiRs. pre-miR-133a-1 and -2 have nearly identical hairpin structures with a few nucleotide differences in their loop regions (Supplementary Figure S3). The same reads can map to both hairpins, thus making the arm abundance of 5′-isomiRs difficult to precisely determine for individual members of miR-133a. In contrast, pre-miR-133a and pre-miR-133b have different sequences and hairpin structures (Supplementary Figure S3B). Of note, such 5′-isomiR abundance variation has been previously indicated for the miR-2 family which have five members in fruitfly (7). Taken together, paralogous genes with substantial structural variations may be subject to altered enzymic processing, leading to different 5′-isomiR arm abundances.

#### Structural features of pre-miRNAs affect 5′-isomiR arm abundance

**Figure 3.**
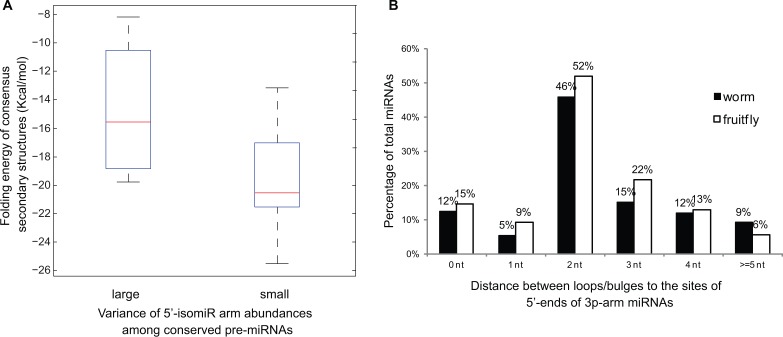
Structural features of pre-miRNAs that affect 5′ end heterogeneities of miRNAs. (**A**) Folding energy of consensus secondary structures, shown in box plot, for two groups of pre-miRNAs conserved across the four animal species considered. Conserved pre-miRNAs with small variance of 5′-isomiR arm abundances among the four species (e.g. let-7 in Table 3) have lower folding energies (box to the right) than those with large variance (e.g. miR-133 in Table 3). The box represents the first and third quartiles; the red line in the middle represents the median value. Whiskers denote the lowest and highest values within 1.53 interquartile range of the first and third quartiles, respectively. (**B**) Distributions of the distances between the sites of 5′ ends of 3p arm major miRNAs to their nearest upstream loops/bulges in fruitfly and worm.

Variation in hairpin structures may give rise to variation in 5′isomiRs. Interestingly, conserved pre-miRNAs with similar 5′-isomiR arm abundances (e.g. let-7 in Table 3) have more conserved secondary structures than those with large variations of 5′-isomiR arm abundances (e.g. miR-124 in Table 3), inferred by the folding energies of consensus structures (T-test *P* < 0.05, Figure 3A). For example, pre-let-7a hairpins in the four species had a consensus structure with a folding energy of –21.1 Kcal/mol, but pre-miR-124 hairpins in the four species had a much higher folding energy of the consensus structure (–10.5 Kcal/mol), indicating that pre-miR-124 hairpins were less conserved across the four species.

Moreover, a recent study has discovered a loop-counting rule that determines the cleavage accuracy of Dicer processing in human and mouse (25). The determinant for accurate Dicer processing is the 2-nt distance between 5′ end of a 3p arm major miRNA and its nearest upstream loop or bulge. Here, we further analysed this structural feature for the pre-miRNA hairpins in fruitfly and worm. Over 46% and 52% of major miRNAs on 3p arms had 5′ ends 2-nt away from their nearest upstream bulges or loops in fruitfly and worm, respectively (Figure 3B). Compared to human (32%) and mouse (33%) (25), a higher percentage of miRNAs following the loop-counting rule may partially account for the overall lower 5′-isomiR percentages in fruitfly and worm (Supplementary Table S6). Particularly, the loop-counting rule is applicable to most hairpins. For example, the major fruitfly dme-miR-133 with the seed ‘UGGUCCC’ is located 2-nt away from the upstream bulge (few 5′-isomiRs), but in human, the 5′-isomiR with the same seed ‘UGGUCCC’ is 3-nt away from the upstream bulge in pre-miR-133a-1/2 hairpins (Supplementary Figure S3B), thus plausibly accounting for a higher 5′ end heterogeneity of miR-133a-1/2 in human. Similar observations were made for miR-124, miR-137, miR-193, miR-210, miR-2, miR-79 and miR87 across species, with miRNAs following the loop-counting rule having lower arm abundances of 5′-isomiRs.

### 5′-isomiRs from non-canonical miRNAs and miRNA-like RNAs

Non-canonical miRNAs may also generate an appreciable amount of 5′-isomiRs (Supplementary Figure S4). Moreover, miRNA-like RNAs from snoRNAs tend to have highly imprecise 5′ ends. Specifically, 10 of the 11 previously identified snoRNA-derived miRNA-like RNAs (28,31) expressed in human skin exhibited high 5′ end heterogeneity with an average of 42.8% of reads as 5′-isomiRs in human skin (Supplementary Figure S4). Furthermore, three subtypes of miRtrons, i.e. the regular, 3′-tailed and 5′-tailed miRtrons, have been currently documented (51). The 5′ ends of miRNA precursors of the regular and 3′-tailed miRtrons are released by RNA exosome during intron debranching (51). A low 5′-hetergeneity was observed for the regular and 3′-tailed miRtrons (Supplementary Figure S4), reflecting an accurate RNA exosome cleavage at the intron splice site ‘GU’. However, the release of pre-miRNAs has not been elucidated for 5′-tailed miRtrons, although the mechanism is independent of Drosha (51). A substantial number of miRNAs on the 5p arms of 5′-tailed miRtrons had a high 5′-heterogeneity (Supplementary Figure S4), indicating an inaccurate enzymic cleavage releasing precursors of 5′-tailed miRtrons. The recent evolutionary origins of many 5′-tailed miRtrons could contribute to imprecise 5′ end processing, as mentioned previously that non-conserved canonical miRNAs tend to have less precise 5′ processing than conserved canonical miRNAs (8).

### Potential functions of 5′-isomiRs

We carried out comprehensive analyses to study the potential functions of 5′-isomiRs in murine miRNA knockout assays, in normal and psoriatic human skin, and in human and mouse cell lines and organs.

#### Potential function of 5′-isomiRs revealed by miRNA knockout assays

**Figure 4.**
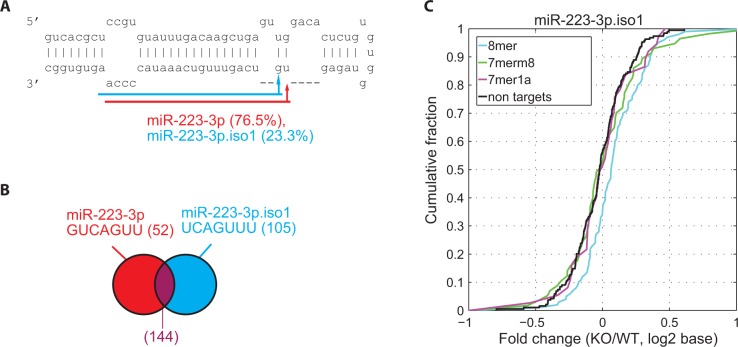
(**A**) The murine mmu-miR-223 hairpin and the most prominent 5′-isomiR. The mature miRNA is highlighted in red and the 5′-isomiR in blue. (**B**) Venn diagram showing the common targets of the major miRNA and the 5′-isomiR, where seed sequence and the number of targets (in parentheses) are shown. The number of exclusive targets of the 5′-isomiR is shown in blue. (**C**) Impact of the 5′-isomiR on mRNA expression variation. Response of mRNAs after deleting mmu-miR-223 gene was determined by the fold change of mRNA expression between knockout (KO) and wild-type (WT) conditions (KO/WT). The *x*-axis corresponds to the fold change of mRNAs in an ascending order. The *y*-axis indicates the portion of mRNAs with their fold changes less than the corresponding value on the *x*-axis. The cumulative distributions of fold changes are plotted for the exclusive targets of mmu-miR-223.iso1. Targets are categorized based on the presence of canonical binding sties on 3′ UTR of mRNAs (8mer, 7mer-m8 and 7mer-1A sites matching the seed regions of 5′-isomiRs and mRNAs with no target sites). Fold-change values of 5′-isomiR exclusive targets (8mer) are larger than those of non-targets, inferred by the cumulative distribution of 8mer targets (the blue cure) drifting to the right away from the non-targets (the black curve). The result is consistent with the expectation that 8mer targets of the 5′-isomiR are more de-repressed than non-targets.

To investigate the functional impact of 5′-isomiRs on mRNA gene regulation, we first examined 5′ heterogeneity of miRNAs using sequencing data and then analysed data of microarray mRNA expression profiling with and without knocking out mmu-miR-223 in murine neutrophils (GSE22004) (21). mmu-miR-223 had a substantial amount of 5′-isomiRs (around 23%) from the 3p arm (Figure 4A), as determined by the sequencing data from murine neutrophils (GSM539851, GSM539852) (52).

In order to evaluate the potential impact of mmu-miR-223.iso1 on mRNA expression, 105 exclusive targets of the 5′-isomiR were identified out of the 249 putative targets (Figure 4B) to avoid the possible intervention of the major miRNA (see Methods section). The comparison of the gene expressions of the exclusive targets and non-targets (mRNAs without target sites of the major miRNA or 5′-isomiR) before and after knocking out mmu-miR-223 showed that mmu-miR-223.iso1 had its exclusive targets of 8mer sites significantly de-repressed (Figure 4C, *P*-value = 7.4 × 10^−^^4^, K-S test, see Methods section), indicating that the exclusive targets with 8mer sites were affected by the 5′-isomiR.

In addition, expression levels and 3′UTR lengths of the exclusive targets were significantly different from that of all non-targets (Supplementary Figure S5A and C, *P*-values < 10-10 from K-S test). To eliminate this difference, a control set, which contained genes that had similar expression levels and 3′UTR lengths as the exclusive targets, was constructed and used in the analysis (see Methods section). The targets in the control set were not statistically significantly different from the original exclusive targets in terms of 3′UTR length and expression level under normal condition (Supplementary Figure S5B and D, K-S test, *P*-values > 0.5). A test using the control set also had a significantly small *P*-value (Supplementary Table S9), indicating that the expression change of the 5′-isomiR exclusive targets was indeed significantly different from that of the non-targets.

The arm abundance of 5′-isomiRs was positively correlated with the effect of the 5′-isomiRs on target repression. For instance, mmu-miR-223 was more abundant than mmu-miR-223.iso1 (77% versus 23%) and had a greater effect on target repression than mmu-miR-223.iso1, reflected by a smaller *P*-value for the exclusive targets of miR-223 than that for miR-223.iso1 (3.5E-09 versus 8.5E-04 on 8mer in Supplementary Table S9). In addition, the mmu-miR-142 hairpin had similar arm abundances between miRNA and 5′-isomiR (45% versus 55%) in dendritic cells (GSM539853). Consistently, mmu-miR-142 and its 5′-isomiR had similar effects on their exclusive targets in the miR-142 knockout data (4.1E-07 versus 5.0E-08 on 8mer in Supplementary Table S9). Moreover, the common targets of major miRNAs and 5′-isomiRs had much smaller *P*-values (Supplementary Table S9), suggesting that miRNAs and 5′-isomiRs had greater effects on the overlapping targets than their exclusive targets.

#### Potential function of 5′-isomiRs in psoriatic and normal human skin

Eighteen 5′-isomiRs (Supplementary Table S10) exhibited significantly differential expression with more than ±2-fold change in at least one of the following comparisons: psoriatic involved skin (PP) versus normal skin (NN), PP versus psoriatic uninvolved skin (PN) and PN versus NN. Most of these 5′-isomiRs had consistent patterns of differential expression with respect to their major miRNAs, which were analysed in our recent study (35). For example, miR-142-3p has a 2.5-fold upregulation in psoriatic lesions (35); consistently, miR-142-3p.iso1 (Supplementary Table S10) was upregulated 2.3-fold in psoriatic involved skin (PP) versus normal skin (NN), suggesting a role of miR-142-3p.iso1 in epidermal inflammation in psoriatic lesions. Interestingly, hsa-miR-203.iso1 had a more pronounced differential expression (i.e. 2-fold upregulation) than hsa-miR-203 (i.e. 1.6-fold change (35)) in PP versus NN. Because miR-203 in mammals has been characterized in skin morphogenesis as a repressor in the suprabasal layers of the epidermis (53,54), the upregulation of hsa-miR-203.iso1 may suggest a role of 5′-isomiRs in psoriasis.

In order to appreciate the potential functions of the 18 DE 5′-isomiRs, their putative targets (see Methods section) were analysed. Overall, about one-third of the putative target genes were not targeted by the corresponding major miRNAs, implying that these 5′-isomiRs and their major miRNAs potentially play different regulatory functions in human skin.

**Figure 5.**
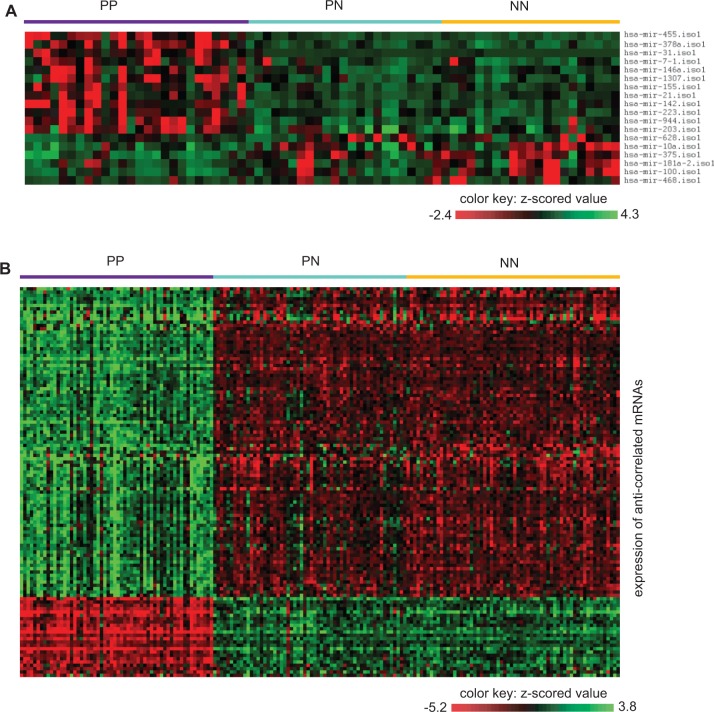
Effect of 5′-isomiRs on the expression of target genes in human psoriatic involved (PP), psoriatic uninvolved (PN) and normal (NN) skin. (**A**) Heat map showing the DE 5′-isomiRs in PP, PN and NN. (**B**) Heap map showing the DE target genes anti-correlated with DE 5′-isomiRs, shown in (A), in PP, PN and NN. The colors in the heat map highlight gene expression of 5′-isomiRs and mRNAs in *z*-scored values.

Among the putative target genes, of particular interest were those that were anti-correlated with the targeting 5′-isomiRs. An additional benefit of focusing on anti-correlated putative targets is that these targets may more likely be genuine targets as previously shown (55). The anti-correlation analysis was done by taking advantage of previously published mRNA profiling data from psoriatic and normal human skin (47,56,57). This analysis resulted in a total of 148 anti-correlated miRNA-mRNA pairs with 110 distinct target genes (21 upregulated and 89 downregulated, Supplementary Table S11). Figure 5 shows the expression patterns of the 18 DE miRNAs and the 110 anti-correlated targets in the psoriatic and normal skin. As shown, these 5′-isomiRs and their targets well characterize the normal and psoriatic skin samples. As a control, we observed a significantly lower number of DE genes among the putative targets of 5′-isomiRs with no significant fold change (T-test, *P* < 0.05), indicating an enrichment of DE target genes in association with DE 5′-isomiRs. Moreover, a GO enrichment analysis (see Methods section) on these DE target genes revealed that the most prominent biological function was regarding ‘positive regulation of gene expression’ and the most significant molecular function was related to ‘transcription factor activity’, suggesting the targeting 5′-isomiRs might have influenced gene expression in human skin by targeting the regulators of gene expression. Taken together, these results suggested that aberrant expression of 5′-isomiRs contributed to the psoriatic pathogenesis.

#### Tissue-specific expression of 5′-isomiRs

It is known that many miRNAs have tissue or cell type specificities (58,59). The arm abundances of 5′-isomiRs across human skin and brain were examined to investigate whether 5′-isomiRs also expressed in a tissue-specific fashion. Indeed, 10 5′-isomiRs had different arm abundance over 20% between skin and brain (chi-square test, *P*-value < 0.05; Supplementary Table S12A). The most prominent example was miR-203-3p.iso1 (46% arm abundance in skin versus 15% in brain). Moreover, miR-133a had a significant difference of arm abundances between human skin and brain (47% in skin versus 25% in brain); this tissue-specific expression pattern persisted in mouse (43% in skin versus 26% in brain).

In addition, seven miRNA hairpins switched dominance between miRNAs and 5′-isomiRs in different tissues (Supplementary Table S12B). For example, the currently annotated hsa-miR-183-5p was more abundant than miR-183-5p.iso1 in skin but less abundant in brain (Supplementary Table S12B). Furthermore, consistent with the previous results (8), dominant 5′-isomiRs as well as miRNAs switched arms under different conditions. Particularly, four 5′-isomiRs, along with their major miRNAs, switched from one arm in human skin to the other arm in brain (Supplementary Table S13). Taken together, whether a small RNA from a miRNA hairpin should be referred to as a miRNA or an isomiR needs to take into consideration the specific organ or tissue of interest.

## DISCUSSIONS

This comprehensive meta-analysis of miRNA isoforms in four animal species was enabled by a large amount of small-RNA data from the Next Generation deep sequencing, particularly that of 67 human skin biopsy samples from our recent study of psoriatic and normal human skin (31,35). Taking advantage of such a large quantity of sequencing data, we were able to scrutinize the heterogeneity of miRNAs to an unprecedented degree of resolution. As a caveat, we note that small-RNA sequencing may be subject to biases due to such factors as non-random adapter ligation and PCR amplification efficiency (60,61).

### Broad existence and conservation of 5′-isomiRs

The majority (>70%) of expressed miRNA hairpins in the four species analysed produced 5′-isomiRs. A large number of 5′-isomiRs appeared in multiple tissues and cell lines, indicating a ubiquitous expression of 5′-isomiRs in an organism. Moreover, orthologous miRNA families across species were able to produce conserved 5′-isomiRs with the same seeds, reflecting a well-conserved mechanism of producing 5′-isomiRs during evolution.

#### Diverse genomic loci of 5′-isomiRs

It has been shown that miRNAs can originate from diverse loci and through a variety of canonical and non-canonical biogenesis pathways (51). In addition to 5′-isomiRs from canonical miRNAs, a substantial number of non-canonical miRNAs such as 5′-tailed miRtrons in human had a high degree of 5′ end heterogeneity. Other types of miRNA-like RNAs, such as snoRNA-derived miRNA-like RNAs, could also host heterogeneous miRNA isoforms in human, indicating diverse genomic origins for 5′-isomiRs. Some previous results have indicated that 5′-isomiRs arise from murine gamma herpesvirus miRNAs, which are recognized as non-canonical miRNAs (62). Particularly, more than one-third of the virus-encoded miRNAs, such as miR-M1-2, have a large number of sequencing reads from the precursor as 5′-isomiRs (12,16).

#### 5′-isomiRs from paralogous miRNAs

Paralogous miRNAs in a family can make it difficult or even impossible to determine the origin of 5′-isomiRs, particularly when paralogs share nearly identical mature or precursor sequences, e.g. miR-124-1/2/3 and miR-133a-1/2. For those paralogous miRNAs and 5′-isomiRs with (nearly) identical sequences, they can be experimentally distinguished if their corresponding precursor sequences are different. For example, if the loop regions of their hairpins differ, they can be separated by PCR assays using step-loop specific primers (63,64). Nevertheless, reads representing 5′-isomiRs appeared in the data that we analysed, supporting the existence of 5′-isomiRs as genuine RNAs. These reads had shifted seed regions different from the annotated major miRNAs. Some paralogous hairpins had prominent structural and sequence changes, and different arm abundances of 5′-isomiRs were observed among such paralogous miRNAs, e.g. miR-133a-1/2 and miR-133b.

### Characteristics of 5′-isomiRs

Previous studies have showed that there is an overall higher degree of 5′ end heterogeneity from the 3p arms than the 5p arms of human miRNA hairpins, implying a higher fidelity of Drosha than Dicer (34). Here, we further observed that the number of conserved miRNAs with high 5′-heterogeneities on the 3p arms is substantially more than that on the 5p arms. Indeed, 39 miRNA hairpins, e.g. the hairpin of miR-203, had high 5′-heterogeneities on the 3p arms but low 5′-heterogeneities on the 5p arms, supporting the previous observation of a higher fidelity of Drosha than Dicer (34). Nevertheless, a few exceptions to this observation exist. For example, miR-411 in human and mouse had more than 40% 5′-isomiRs on the 5p arm versus <1% on the 3p arm.

Interestingly, for those hairpins with high 5′-heterogeneities on the 3p arms, 3′-isomiRs had similar arm abundances on both the 5p and 3p arms (data not shown), which was contrary to the expectation that more 5′-heterogeneities on the 3p arms are in parallel with more 3′-heterogeneities on the 5p arms. The underlying mechanism accounting for 5′-heterogeneities on either 5p or 3p arms remains to be further investigated.

#### Seed shifting and pre-miRNA structures

miRNA seed regions have been implicated to have functional significance (2). Seed shifting has been observed between miRNA paralogous genes in *Drosophila* (13) and miRNA orthologues across different species such as *Drosophila* and *Tribolium casteneum* (50,65,66). We observed that miRNA orthologues (miR-10, miR-133, miR-137 and miR-79 in Table 3) swapped major miRNAs and 5′-isomiRs and had largely different 5′-isomiR arm abundances across human, mouse, fruitfly and worm*.*

Recent studies on human and mouse reveal that properties on pre-miRNA structures can affect the accuracy of Dicer processing (24,25,67,68). The additional analysis across the four species in the current study supported the findings in (25) which helped explain why miRNAs from the 3p arms of hairpins were more heterogeneous than from the 5p arms. Moreover, we observed pre-miRNA structures were strongly associated with the seed shifting observed among orthologous miRNAs. Particularly, length-counting rules based on pre-miRNA structures may explain the phenomena of seed shifting and heterogeneity of orthologous miRNAs between species, i.e. miRNA hairpins following the loop-counting rule have lower arm abundances of 5′-isomiRs.

#### Tissue-specificity of 5′-isomiRs

Comparison of 5′-isomiR expression profiles in human skin and brain revealed significant tissue specificity of 5′-isomiRs. Similar observations have been made on 3′-isomiRs in *D. melanogaster*, which are DE across different developmental stages and tissues (10). Besides, a recent study has showed that Dicer can tune the cleavage sites on miR-307a precursor in fruitfly by associating with various loqs protein isoforms, leading to the generation of 5′ end miRNA isoforms of various lengths (23). More interestingly, evidence supports that the relative abundance of loqs isoforms varies widely among tissues and developmental stages (23). Taken together, it suggests that Dicer and its partner proteins have the potential to regulate the relative abundance of 5′-isomiRs in a spatiotemporal fashion.

### Functional impact of 5′-isomiRs

Several miRNA variants have been implicated in other diseases, including Huntington’s disease (33) and cancer (69). In a model of mouse leukemia, miRNA variants of mmu-miR-10a, mmu-miR-155, mmu-miR-27a, mmumiR-27c, mmu-let-7a and mmu-miR-222 are found DE across disease and normal conditions (17). More interestingly, mmu-miR-223, in which 5′-isomiRs accumulate to a level comparable to the major form, is highly downregulated in tumor cells (17). 5′-isomiRs have also been noticed on both the 5p and 3p arms of hsa-miR-142 hairpin in T cells where miR-142 is the most highly expressed miRNA in naive T cells (70).

Some of the 5′-isomiRs detected in the current study may also have disease related functions. Of particular importance are those 5′-isomiRs that exhibit abnormal expression in psoriatic skin. One prominent example is hsa-miR-203 in which 5′-isomiRs accumulated to more than 40% reads and were more than 2-fold upregulated in psoriasis. hsa-miR-203, found in suprabasal layers of the epidermis, limits the proliferative potential of keratinocytes and establishes a well-defined boundary between proliferating and terminally differentiating keratinocytes (53,54). hsa-miR-203-3p has been found to target, among many other mRNAs, Δp63 that has multiple functions during skin development (53,54). hsa-miR-203-3p and the most abundant 5′-isomiR, hsa-miR-203-3p.iso1, share only about half (57.3%) common targets, implying their potentially distinct functions. Given that hsa-miR-203-3p.iso1 was high expressed and upregulated in psoriatic skin, it might very well play a critical role in psoriatic pathogenesis. Another 5′-isomiR, miR-142-3p.iso1 was upregulated by 2.3-fold in psoriatic skin (Supplementary Table S10) and shown to repress targets distinct from the targets of miR-142-3p. Our recent study has revealed the epidermal infiltration of the hematopoietic-specific miR-142-3p in psoriatic lesions (35). Taken together, the high abundance and upregulation of 5′-isomiRs of hsa-miR-203 and hsa-miR-142 may indicate their functional roles in inflammatory and hyperproliferative phenotype of psoriatic lesions.

## CONCLUSIONS

This meta-analysis of miRNA isoforms has deepened our understanding of the broad existence and conservation of 5′-isomiRs in the four model species. Our results revealed that 5′-isomiRs from the 3p arms of mammalian miRNA hairpins had an overall higher 5′ end heterogeneity than from the 5p arms. Besides, the variation of hairpin structures was strongly associated with the variation of 5′-isomiRs among paralogs in an organism and among orthologs across species. The analysis has revealed robustness and plasticity of miRNA mediated post-transcriptional gene regulation. Though they shared a substantial amount of common target genes, many 5′-isomiRs and major miRNAs also had their distinct exclusive target genes and thus were likely to have distinctive regulatory functions. In light of the function of many miRNAs (e.g. miR-203 and miR-142) in skin and psoriasis, DE 5′-isomiRs (such as miR-203-3p.iso1 and miR-142-3p.iso1) were likely to play a role in psoriasis pathogenesis. In short, the current study represented critical first steps toward fully characterizing causes and functions of miRNA isoforms.

## SUPPLEMENTARY DATA

Supplementary Data are available at NAR Online.

Supplementary Data
