# Supplementary material for: A meta-analysis revealed insights into the sources, conservation and impact of microRNA 5′-isoforms in four model species
Source: Nucleic Acids Res. 2013 Oct 30;42(3):1427–41. doi: 10.1093/nar/gkt967 (PMC3919606; doi:10.1093/nar/gkt967)
Supplement: Supplementary Data [file supp_42_3_1427__index.html]

A meta-analysis revealed insights into the sources, conservation and impact of microRNA 5′-isoforms in four model species — A meta-analysis revealed insights into the sources, conservation and impact of microRNA 5′-isoforms in four model species — Supplementary Data 

# A meta-analysis revealed insights into the sources, conservation and impact of microRNA 5′-isoforms in four model species

## Supplementary Data

files

**Files in this Data Supplement:**

- Supplementary Data - docx file
- Supplementary Data - xlsx file
