# Supplementary material for: A meta-analysis revealed insights into the sources, conservation and impact of microRNA 5′-isoforms in four model species
Source: Nucleic Acids Res. 2013 Oct 30;42(3):1427–41. doi: 10.1093/nar/gkt967 (PMC3919606; doi:10.1093/nar/gkt967)
Supplement: Supplementary Data [file supp_gkt967_nar-00611-y-2013-File008.docx]

**Supplementary Figure S1.** Comparisons of arm abundances of 5’-isomiRs across species (black arrows) and the comparisons across tissues (red arrows) that were performed in the study. The blue arrow indicates the comparison of tissue specificities in human and mouse.

**Supplementary Figure S2.** (**A**) Sequence conservation of miR-10 among human, mouse and fruitfly with major miRNAs annotated in purple, showing that the 5’-isomiR and major miR-10a-5p swap across these species. (**B** and **C**) Folding structures of miR-10 hairpins in human, mouse and fruitfly, showing 5’-heterogeneity of miR-10-5p among human, mouse and fruitfly*.*

**Supplementary Figure S3.** (**A**) Sequence conservation of pre-miR-133 in human and fruitfly with major miRNAs annotated in red, showing that the change of major miR-133-3p between human and fruitfly*.* (**B**) Folding structures of pre-miR-133 hairpins in human and fruitfly. pre-miR-133a-1 and -2 are nearly identical with a few nucleotide differences, represented by the dots in the loop region. The distances between the miRNAs with the seed “UGGUCCC” and their nearest upstream loop/bulge are marked in each hairpin.

**Supplemental Figure S4**. Distribution of 5’-isomiR arm abundances of noncanonical miRNAs and miRNA-like RNAs. The numbers of 5’-isomiR reads and arm abundances were counted by human psoriasis datasets. The 5’-isomiR arm abundances were categorized into 5’ tailed miRtrons (green), 3’-tailed miRtrons (black), regular miRtrons (purple), snoRNA-derived (red) and tRNA-derived (blue) miRNA-like RNAs, respectively.


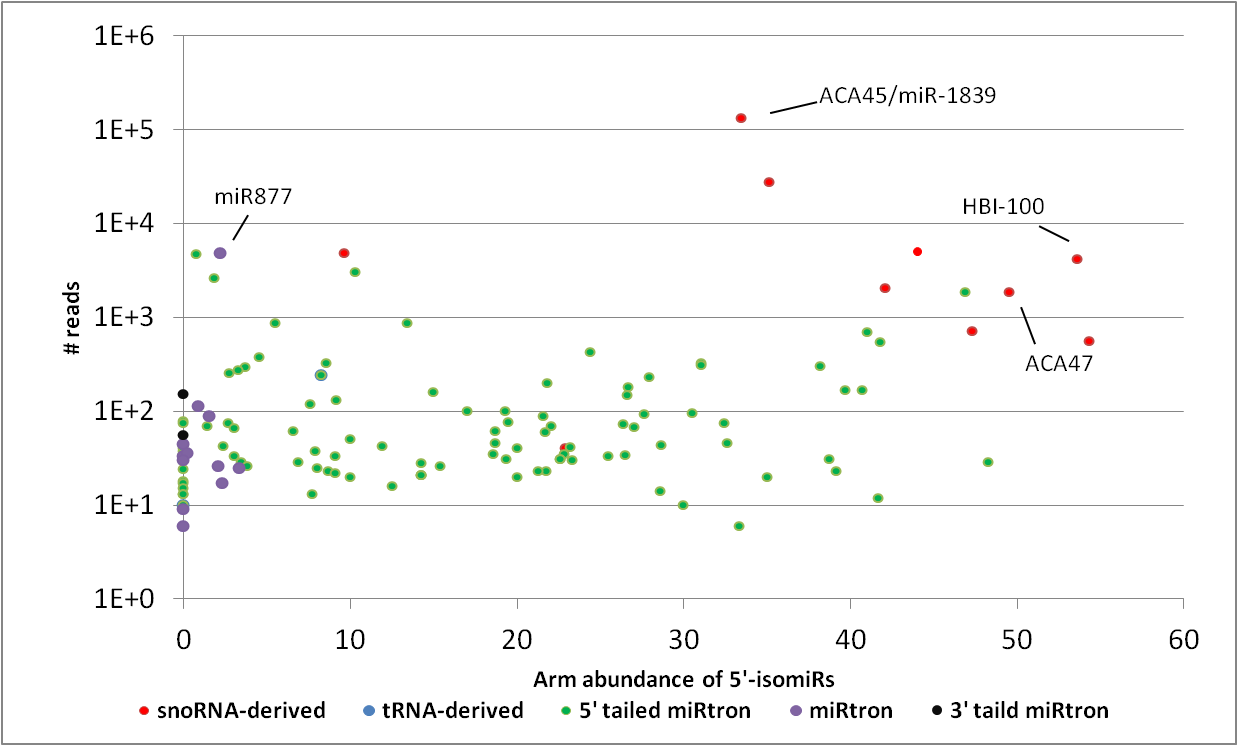


**Supplementary Figure S5.** Comparisons of gene expression levels and 3’ UTR lengths between mmu-miR-142.iso1 targets and control genes. (**A**) Comparison of gene expression levels between target mRNAs and all non-target genes. The x-axis corresponds to the gene expression levels of mRNAs (raw robust multi-array average value) in an ascending order. The y-axis indicates the portion of mRNAs with their expression levels less than a corresponding value of x-axis. The cumulative distributions of gene expression are plotted for the 5’-isomiR targets and for all non-target mRNAs, respectively. The two distributions were significantly different as determined by p-value <10^-10^, K-S test. (**B**) Comparison of gene expression levels between target mRNAs and a control set randomly collected from the non-target genes whose expression levels are similar to target genes (within a range of 20 expression value; the two distributions were not significantly different as determined by p-value >0.5, K-S test). (**C**) Comparison of 3’ UTR lengths between target mRNAs and all non-target genes (p-value <10 ^-20^, K-S test). (**D**) Comparison of 3’ UTR length between target mRNAs and a control set randomly sampled from the non-target genes whose 3’UTR lengths are similar to the target genes (within a range of 50-nt; p-value >0.5, K-S test)
